# Not all “*Candida*” are “*Candida*”: *Nakaseomyces glabratus* (H.W. Anderson) Sugita & M. Takask. 2022 onychomycosis and the need for rapid diagnostic

**DOI:** 10.1093/femsle/fnaf107

**Published:** 2025-10-03

**Authors:** Mirko Benvenuti, Giulia Gasparini, Antonino Torino, Emanuele Claudio Cozzani

**Affiliations:** Section of Dermatology, Department of Health Sciences (DISSAL), University of Genoa,Via A. Pastore 1, 16132 Genoa, Italy; Section of Dermatology, Department of Health Sciences (DISSAL), University of Genoa,Via A. Pastore 1, 16132 Genoa, Italy; IRCCS Ospedale Policlinico San Martino, clinica di Dermatologia Sociale, Largo Rosanna Benzi 10, 16132 Genoa, Italy; Section of Dermatology, Department of Health Sciences (DISSAL), University of Genoa,Via A. Pastore 1, 16132 Genoa, Italy

**Keywords:** nakaseomyces glabratus, candida, onychomycosis, infection, nail

## Abstract

Onychomycosis is a common nail disorder typically caused by dermatophytes, with *Candida* species and non-dermatophytic molds being less frequent agents. Nakaseomyces glabratus (formerly *Candida glabrata*) is a rare cause of nail infections, notable for its intrinsic resistance to azoles and diagnostic challenges. We report a case of chronic nail discoloration, thickening, and fragility initially misdiagnosed as psoriasis. Microscopy and culture, followed by selective medium analysis, confirmed *N. glabratus* infection. This case emphasizes the importance of considering uncommon yeast species and highlights both the need for accurate identification to guide therapy and the possible misdiagnosis with other pathologies. Awareness and proper diagnostic approaches are essential for timely and effective management of such rare fungal nail infections.

## Teaser

Onychomycosis can rarely involve Nakaseomyces glabratus,highlighting diagnostic challenges, misidentification risks, and the importance of accurate detection for effective treatment.

## Introduction

Fungi are capable of causing harm to humans through three main mechanisms: infections (mycoses), toxin-mediated effects (mycotoxicoses), and toxic reactions due to ingestion of poisonous mushrooms (mycetism) (Benvenuti et al. 2024). Among these, fungal infections represent the most clinically significant cause of morbidity, ranging from superficial skin and nail diseases to severe systemic mycoses. While dermatophytes are traditionally recognized as the leading agents of superficial fungal infections, in recent decades an increasing number of non-dermatophytic molds and yeasts have been identified as opportunistic pathogens (Benvenuti, Burlando and Cozzani 2025, Ye at al. 2025 et al. 2025).

Onychomycosis is the most frequent fungal infection of the nails, accounting for approximately 50% of all nail disorders (Zaraa et al. 2024, Merlo et al. 2025). The majority of cases are caused by dermatophytes, followed by yeasts of the genus *Candida* and, less frequently, non-dermatophytic molds (Benvenuti and Cozzani 2025). Although often regarded as a benign condition, onychomycosis can significantly impact quality of life, leading to pain, discomfort, cosmetic concerns, and, in certain populations, an increased risk of secondary bacterial infections.

While *Candida albicans* remains the most common yeast implicated in nail infections, other non-*albicans Candida* species have increasingly been recognized as emerging pathogens in recent years (Zhang et al. 2024). Among them, *Nakaseomyces glabratus* (formerly classified as *Candida glabrata*) is of particular interest. *N. glabratus* is a commensal yeast of the human gastrointestinal and genitourinary tracts and is best known for its role in invasive candidiasis, candidemia, and mucosal infections. Unlike *C. albicans, N. glabratus* lacks the ability to form true hyphae and is intrinsically less virulent; however, it compensates through remarkable adaptability, rapid acquisition of antifungal resistance, and persistence in host tissues (Duggan and Usher 2023, Katsipoulaki et al. 2024).

In the context of nail infections, *Nakaseomyces glabratus* is rarely reported. This is partly due to the ongoing taxonomic confusion, as many authors still refer to the older binomial *Candida glabrata*, and partly because in superficial infections such as onychomycosis, species-level identification is often not pursued in depth (Jayatilake wt al. 2009 et al. 2009). This lack of detailed investigation is particularly evident for yeasts, which are frequently grouped under the generic diagnosis of *onychomycosis due to Candida* without further species differentiation.

Even though the literature contains only isolated case descriptions, suggesting that the true prevalence may be underestimated also due to challenges in routine diagnosis (Vasconcellos et al. 2013). Standard culture techniques may misidentify or fail to detect *N. glabratus*, and molecular methods are not always available in daily clinical practice. Furthermore, the therapeutic management of these infections is complicated by the species’ reduced susceptibility to azoles, particularly fluconazole, which is widely used as first-line therapy in fungal nail infections (Naskar et al. 2025; Duggan and Usher 2023).Consequently, due to the frequent occurrence of intrinsic resistance among certain fungal species, it is often necessary to resort to combined antifungal therapy to achieve optimal therapeutic outcomes (Campitelli et al. 2017).

The rarity of onychomycosis caused by *N. glabratus* underscores the importance of reporting new cases. Such reports not only broaden our understanding of the clinical spectrum associated with this organism but also highlight the diagnostic and therapeutic difficulties that clinicians may encounter (Ohn et al. 2020, Jabrodini et al. 2025). Here, we describe a rare case of onychomycosis due to *Nakaseomyces glabratus*, focusing on the clinical presentation, diagnostic process and treatment outcome. Our aim is to contribute to the limited but growing body of evidence regarding this unusual presentation and to emphasize the need for heightened awareness and accurate identification of uncommon fungal pathogens in nail infections.

## Case presentation

**Figure 1.**
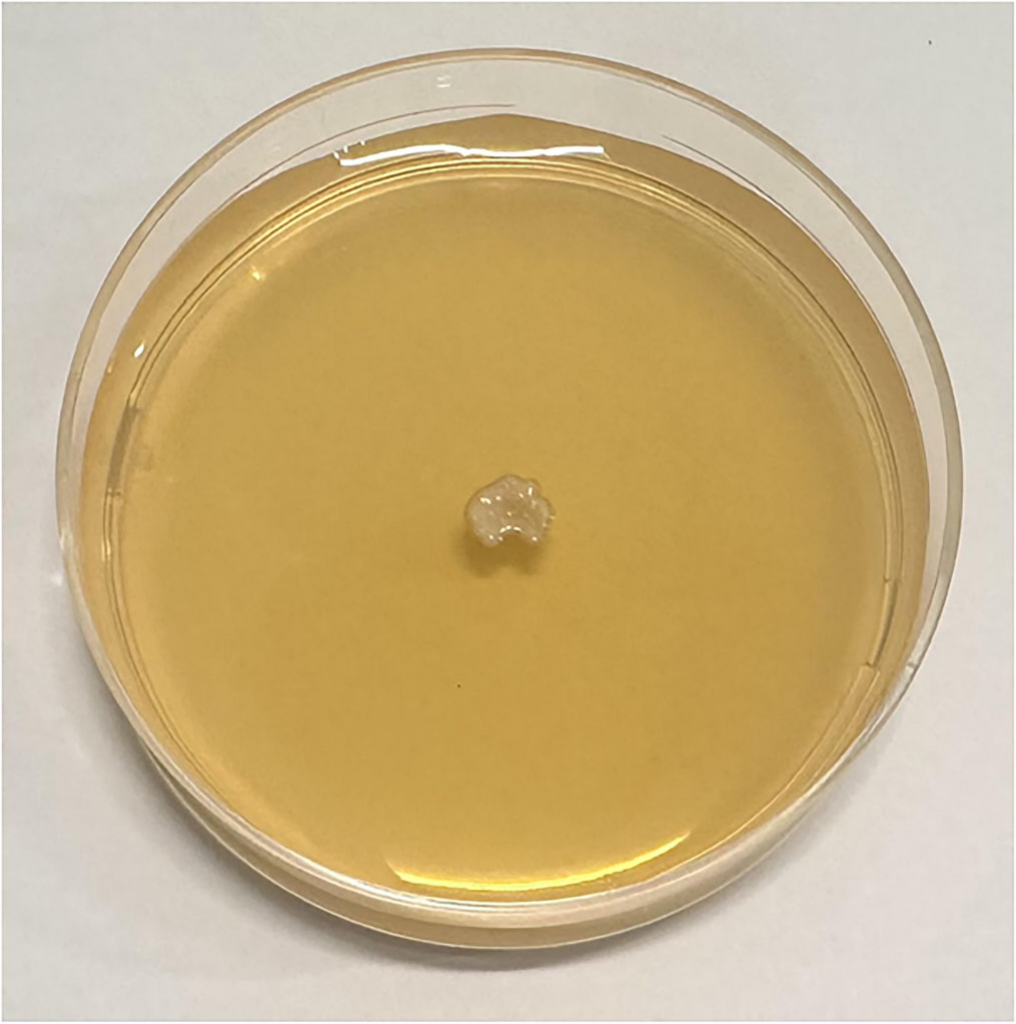
Colony morphology of *Nakaseomyces glabratus* grown on Sabouraud dextrose agar after 48 hours of incubation at 30°C. The isolate displays markedly reduced colony size compared to other yeasts, with a slow growth rate evident at this stage. Colonies are characteristically smooth, convex, and exhibit a glossy surface, in contrast to the typically larger, opaque, and more matte colonies produced by other *Candida* species. These distinctive macroscopic features may assist in the presumptive differentiation of *N. glabratus* from more common etiologic agents of onychomycosis.

A 62-year-old female patient was referred to our dermatology clinic with a multi-year history of progressive nail abnormalities affecting the first three fingers of her left hand. She reported gradual, persistent changes including nail discoloration, onycholysis, increased fragility, and recurrent episodes of paronychia. The patient described these changes as increasingly noticeable during daily activities, leading to both functional discomfort and aesthetic concern. Over time, the nails became more brittle, irregular in shape, and prone to minor fissures, complicating routine hand use Fig. 1

The patient had previously been diagnosed with chronic nail psoriasis and had undergone multiple courses of topical corticosteroids and keratolytic treatments. These therapies provided no improvement, and she noted a gradual worsening of the nail condition, with the involvement slowly extending to adjacent nails.

Her past medical history was notable only for a family history of cardiovascular diseases. She denied diabetes mellitus, immunosuppressive therapy, peripheral vascular disease, or other systemic comorbidities. There was no personal or family history of psoriasis or other chronic dermatologic conditions. She reported no history of trauma to the affected nails, frequent exposure to communal water facilities, habitual nail-biting, prolonged glove use, or recent manicures or artificial nail application.

On physical examination, the affected nails demonstrated diffuse brownish discoloration, thickening, increased curvature, and irregular surfaces. Partial onycholysis was observed in all three nails, while the surrounding periungual skin showed mild erythema and tenderness consistent with cyclical paronychia. No maceration, purulent discharge, or secondary bacterial infection was present at the time of evaluation. The lunulae were partially visible, and the rest of the nails on both hands and feet appeared clinically normal.

Given the chronicity of the nail changes, the atypical presentation, and the lack of response to prior therapies, a fungal etiology was suspected. Nail scrapings were obtained for direct microscopy and culture to identify potential pathogens. The case was documented carefully, considering both the unusual presentation and the risk of rare fungal involvement in refractory nail disorders.

## Material and methods

Nail specimens were obtained from the affected patients by blade scraping of the dystrophic nail surface. Samples were inoculated onto Sabouraud Dextrose Agar (SDA) prepared in the laboratory with the following composition per 0.5 L of distilled water: agar 10 g, peptone 10 g, D(+)glucose 5 g, chloramphenicol 0.1 g, and cycloheximide 0.5 g. In addition, Dermatophyte Test Medium (DTM) was employed (Merck Italy, Via Monte Rosa 93–20 149, Milan). The inoculated plates were incubated at 30°C under high relative humidity with 12-hour alternating light/dark cycles. Microscopic examination of the cultures was performed using direct sampling and staining with lactophenol blue. or final species-level identification, CHROMagarTM Candida Plus plates were purchased Chromagar 4 place du 18 juin 1940 75 006 Paris—France. Colony tested on Chromagar have been grown at 37°C for 24 h before species level identification.

## Results

**Figure 2.**
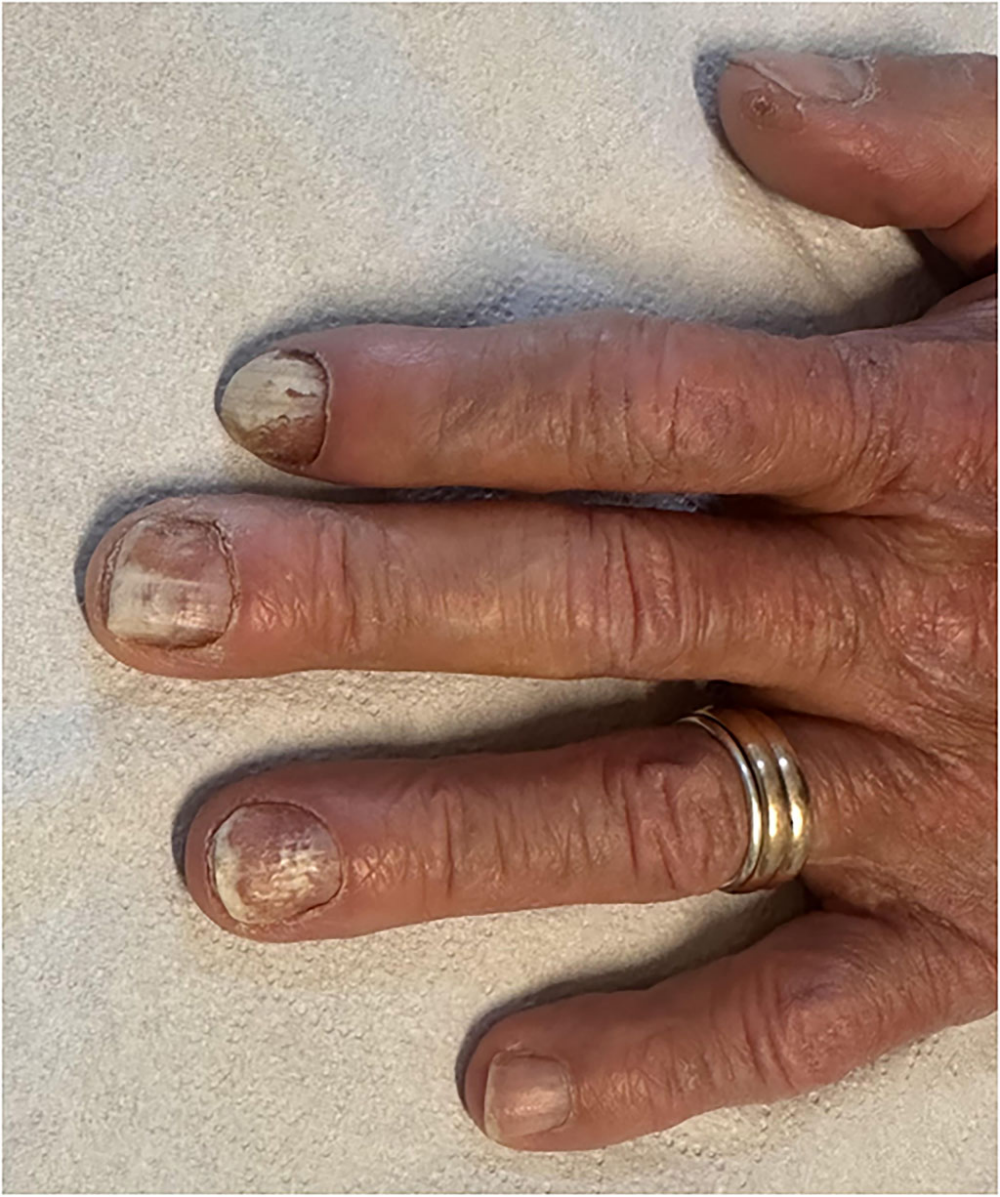
Clinical presentation of yeast onychomycosis involving the index, middle, and ring fingernails. The affected nails display a dull whitish discoloration with irregular surface changes, consistent with yeast-related nail infection. On the index finger, periungual swelling is clearly evident, reflecting recurrent episodes of paronychia, which have contributed to inflammation and distortion of the surrounding nail folds.

Direct microscopic examination of nail scrapings with potassium hydroxide (KOH) preparation revealed numerous small yeast cells, approximately 1–5 μm in diameter, with rounded to ovoid morphology. Notably, no formation of true hyphae or pseudohyphae was observed, a finding consistent with the typical morphology of *Nakaseomyces glabratus*. No dermatophyte structures such as septate hyphae or arthroconidia were detected Fig. 2.

**Figure 3.**
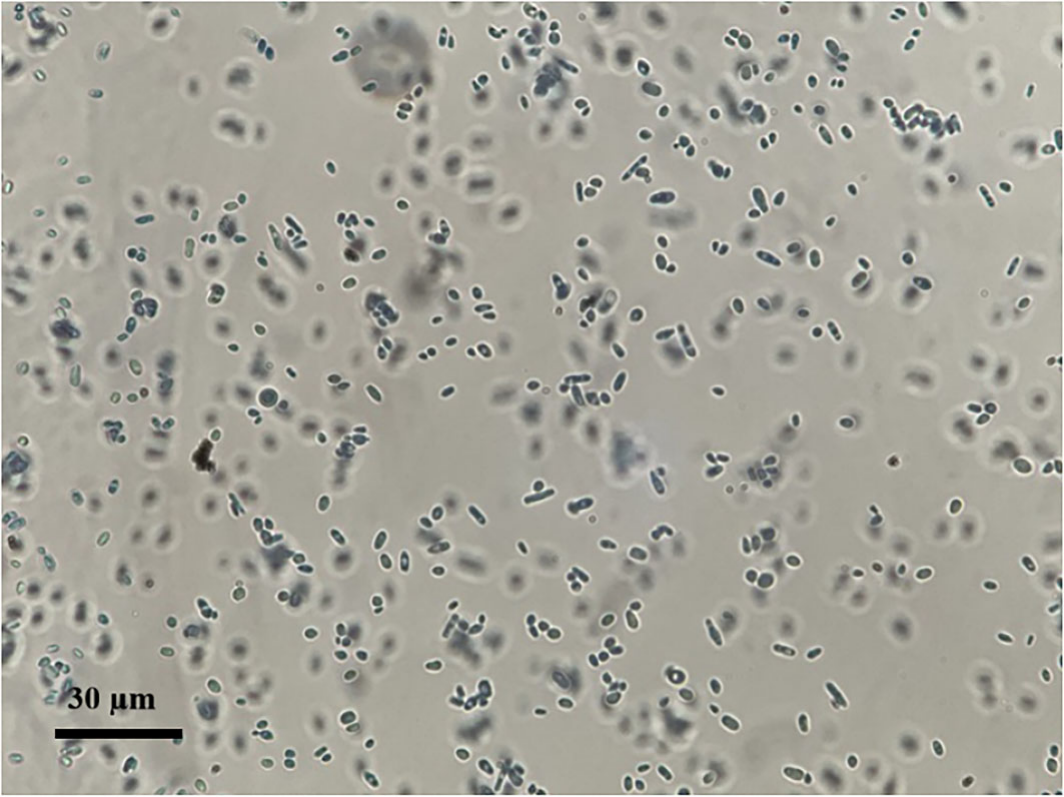
Microscopic appearance of Nakaseomyces glabratus (formerly Candida glabrata) stained with lactophenol cotton blue. The yeast cells measure approximately 1–5 μm in diameter and exhibit a morphology ranging from round to ovoid and occasionally elongated forms. Unlike many other pathogenic yeasts, N. glabratus does not produce true hyphae or pseudohyphae, a distinctive feature that aids in its microscopic differentiation from other fungal species.

Primary cultures on Sabouraud dextrose agar (SDA) demonstrated visible growth after four days of incubation at 30°C, producing smooth, cream-colored colonies with a yeast-like appearance. The relatively slow but consistent growth pattern, coupled with the absence of filamentous structures, supported the suspicion of a yeast Fig. 3.

**Figure 4:**
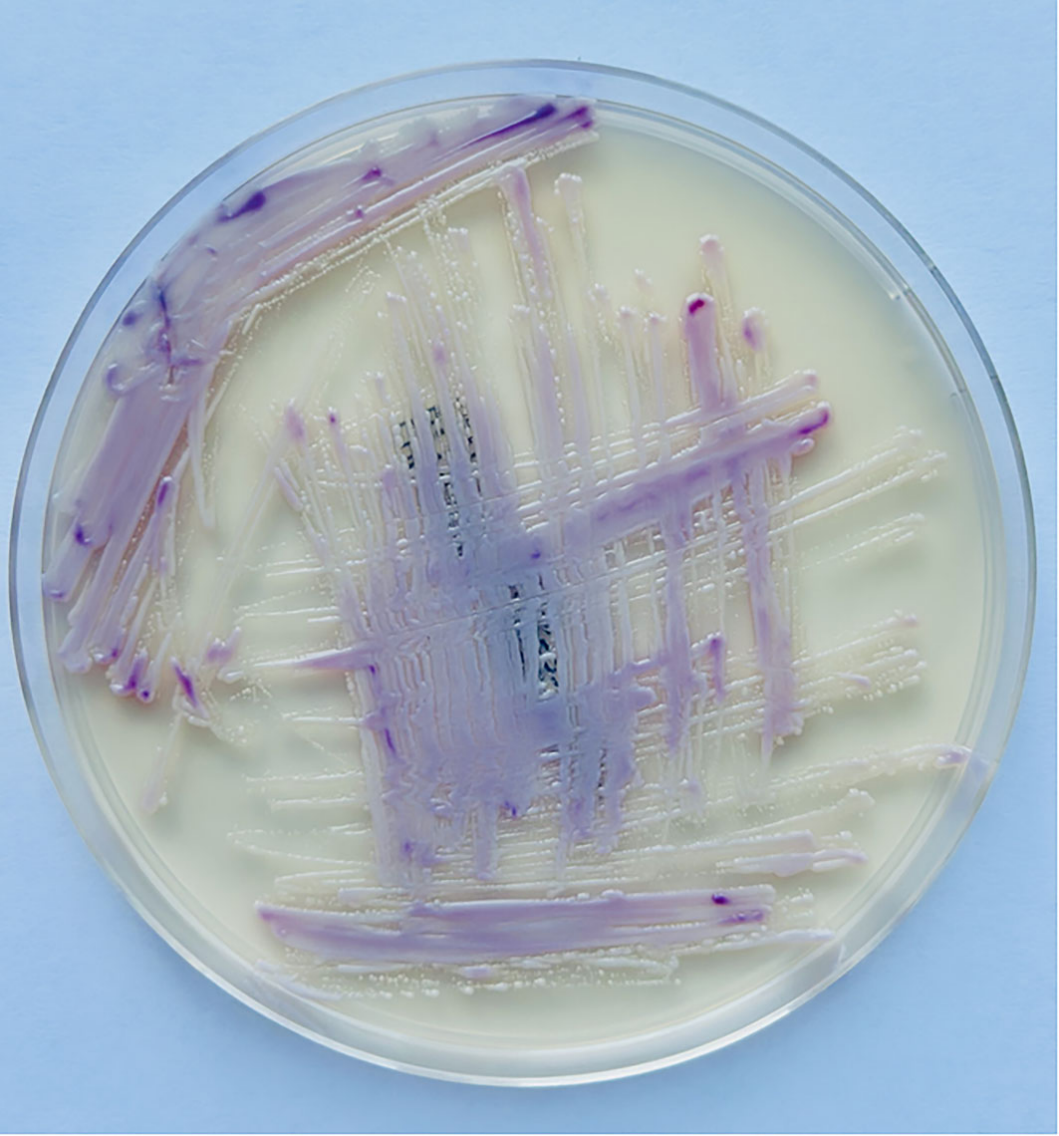
Growth of *Nakaseomyces glabratus* (formerly *Candida glabrata*) on CHROMagar™ Candida Plus. After incubation, the yeast colonies display the characteristic smooth texture and uniform, glossy surface, accompanied by a distinctive mauve coloration. These phenotypic traits are consistent with the selective and differential properties of the medium, allowing the presumptive identification of *N. glabratus* and differentiation from other *Candida* species.

For further identification, the isolate was subcultured onto CHROMagar™ Candida Plus and incubated at 37°C. After incubation, colonies displayed a distinctive mauve coloration with a smooth and glossy surface, a highly characteristic phenotype of *N. glabratus* (formerly *Candida glabrata*) Fig. 4 This colony morphology provided reliable differentiation from other yeasts, which typically produce colonies of differing hues such as green, blue, or white on the same medium.

No dermatophytes or non-dermatophytic molds were detected in parallel cultures, confirming *N. glabratus* as the sole etiological agent responsible for the infection.

## Discussion

*Nakaseomyces glabratus* (formerly *Candida glabrata*) is an uncommon etiological agent of onychomycosis, and its isolation in nail infections remains rare in clinical practice. Traditionally, onychomycosis is primarily attributed to dermatophytes, with *Candida* species and non-dermatophytic molds representing less frequent causes. However, recent reports suggest that rare yeast pathogens, including *N. glabratus*, are increasingly being recognized as responsible for refractory or atypical cases of nail and cutaneous fungal infections (Naskar et al. 2025, Ye et al. 2025). This trend may reflect both improved diagnostic techniques and a growing population of patients with predisposing factors, such as advanced age, comorbidities, or previous antifungal exposure (.Salmanton-García 2024)

In our case, the identification of *N. glabratus* was facilitated by the use of chromogenic media, which allowed rapid presumptive recognition based on the characteristic mauve coloration and smooth, glossy colony morphology. The decision to proceed with subculture on chromogenic medium was made after primary growth on Sabouraud dextrose agar revealed small yeast cells of variable rounded to ovoid shape, with no evidence of true hyphae or pseudohyphae. This microscopic profile was not fully compatible with typical *Candida* species, thereby prompting further investigation through the use of a chromogenic medium to achieve a more reliable differentiation. Microscopically, the yeast cells were small, ranging from 1 to 5 μm, with rounded to ovoid shapes, and notably did not form true hyphae or pseudohyphae. Such features can lead to underdiagnosis if reliance is placed solely on conventional microscopy, highlighting the importance of careful laboratory evaluation in cases unresponsive to standard therapy.

Although *N. glabratus* is well known for its role in mucosal and invasive infections, its occurrence in nail disease is exceptional. Its intrinsic reduced susceptibility to azole antifungals poses additional therapeutic challenges, as empirical treatments may be ineffective. Recognizing rare yeast pathogens in onychomycosis is therefore crucial not only for accurate diagnosis but also for guiding targeted antifungal therapy, minimizing treatment failure, and reducing the risk of chronic infection.

Overall, this case underscores that careful laboratory investigation, including culture on chromogenic media and attentive microscopic examination, can reveal uncommon pathogens that may otherwise go undetected. Increasing awareness of these rare yeast infections is essential, particularly as evidence suggests their prevalence in both onychomycosis and systemic mycoses is on the rise. Reporting such cases contributes to a better understanding of the epidemiology of uncommon fungal pathogens and supports the development of appropriate diagnostic and therapeutic strategies.

## Conclusion

The use of chromogenic selective media represents a valuable tool in the rapid identification of yeast infections in clinical practice. These media allow for presumptive recognition of different species based on distinctive colony coloration and morphology, thereby expediting the diagnostic process. Such rapid differentiation is particularly important when dealing with uncommon or emerging yeast pathogens, which may otherwise be overlooked or misclassified using conventional methods alone (Ghelardi et al. 2008).

In recent years, rare and diagnostically challenging fungal pathogens have been increasingly encountered in both superficial and systemic infections. Their rising prevalence can be attributed to multiple factors, including the widespread use of antifungal agents, aging populations, and the growing number of immunocompromised patients (Duan et al. 2025,Wahab et al. 2025,,). In this context, the early and accurate identification of unusual yeasts is critical, as therapeutic .recognition may result in inappropriate treatment, prolonged infection, and potential complications.

Therefore, the incorporation of chromogenic selective media into routine diagnostic workflows not only enhances laboratory efficiency but also contributes directly to patient care (Mulet Bayona et al. 2022, Bloch et al. 2025). By enabling clinicians to quickly discriminate between common and rare yeasts, these tools support timely, targeted therapeutic interventions, ultimately improving clinical outcomes in cases where standard diagnostic approaches might fall short.
